# Cross-cultural adaptation and validation of the VISA-A questionnaire for German-speaking Achilles tendinopathy patients

**DOI:** 10.1186/1471-2474-10-134

**Published:** 2009-10-30

**Authors:** Heinz Lohrer, Tanja Nauck

**Affiliations:** 1 Institute for Sports Medicine, Otto-Fleck-Schneise 10, D-60528 Frankfurt am Main, Germany

## Abstract

**Background:**

Achilles tendinopathy is the predominant overuse injury in runners. To further investigate this overload injury in transverse and longitudinal studies a valid, responsive and reliable outcome measure is demanded. Most questionnaires have been developed for English-speaking populations. This is also true for the VISA-A score, so far representing the only valid, reliable, and disease specific questionnaire for Achilles tendinopathy. To internationally compare research results, to perform multinational studies or to exclude bias originating from subpopulations speaking different languages within one country an equivalent instrument is demanded in different languages. The aim of this study was therefore to cross-cultural adapt and validate the VISA-A questionnaire for German-speaking Achilles tendinopathy patients.

**Methods:**

According to the "guidelines for the process of cross-cultural adaptation of self-report measures" the VISA-A score was cross-culturally adapted into German (VISA-A-G) using six steps: Translation, synthesis, back translation, expert committee review, pretesting (n = 77), and appraisal of the adaptation process by an advisory committee determining the adequacy of the cross-cultural adaptation. The resulting VISA-A-G was then subjected to an analysis of reliability, validity, and internal consistency in 30 Achilles tendinopathy patients and 79 asymptomatic people. Concurrent validity was tested against a generic tendon grading system (Percy and Conochie) and against a classification system for the effect of pain on athletic performance (Curwin and Stanish).

**Results:**

The "advisory committee" determined the VISA-A-G questionnaire as been translated "acceptable". The VISA-A-G questionnaire showed moderate to excellent test-retest reliability (ICC = 0.60 to 0.97). Concurrent validity showed good coherence when correlated with the grading system of Curwin and Stanish (rho = -0.95) and for the Percy and Conochie grade of severity (rho 0.95). Internal consistency (Cronbach's alpha) for the total VISA-A-G scores of the patients was calculated to be 0.737.

**Conclusion:**

The VISA-A questionnaire was successfully cross-cultural adapted and validated for use in German speaking populations. The psychometric properties of the VISA-A-G questionnaire are similar to those of the original English version. It therefore can be recommended as a sufficiently robust tool for future measuring clinical severity of Achilles tendinopathy in German speaking patients.

## Background

Achilles tendinopathy is the most common differential diagnosis of posterior heel pain [1-4]. Achilles tendinopathy is the predominant overuse injury in runners with a prevalence of 10% [5] and an incidence of 0.01/1000 km [6].

The worth of conservative and operative treatment options is still under debate [7]. To further investigate these issues in transverse and longitudinal studies a valid, responsive, and reliable outcome measure is demanded. For this reason an international group of experts from the Victorian Institute of Sports Assessment developed the VISA-A (the second A stands for "Achilles tendon") questionnaire [8].

"The VISA-A is an easily self-administered questionnaire that evaluates symptoms and their effect on physical activity. It can be used to compare different populations with Achilles tendinopathy, and facilitate comparisons between studies. It can be used to determine the patient's clinical severity and provide a guideline for treatment as well as for monitoring the effect of treatment [9]." Pain (questions 1-3), function (questions 4-6), and activity (questions 7 and 8) are the domains under consideration [8]. Maximum score of each item one to seven is 10, while question 8 yields a maximum of 30. Consequently a summed maximum score of 100 represents an asymptomatic person [8].

The VISA-A questionnaire represents the only valid, reliable, and disease specific score to measure the condition of the Achilles tendon. Meanwhile it achieved wide acceptance [9-16]. However, as most questionnaires it has been developed for an English-speaking population. To internationally compare research results, to perform multinational studies or to minimize bias originating from subpopulations speaking different languages within one country a unique protocol of forward and back translations and cultural adaptations as well as "verification of the scaling requirements (item performance, item weights) and validation of and establishing normative values for the new version" are required [17]. This procedure has already been performed and is published for the Swedish [9] and Italian [12] version of the VISA-A questionnaire. Cross-cultural adaptations to Spanish, Portuguese, and Flemish language have been done (Karim Khan personal communication, 2009) but were not reported in journals available in Medline/PubMed. The aim of this study was therefore to cross-culturally adapt and validate the VISA-A questionnaire for German-speaking Achilles tendinopathy patients.

## Methods

The study was approved by the local ethics committee.

According to the "guidelines for the process of cross-cultural adaptation of self-report measures" the VISA-A score was cross-culturally adapted into German (VISA-A-G) in six steps [17].

Step I: Initial translation from English to German (forward translation) was performed by three independent translators with German mother tongue. An orthopaedic surgeon specialised in foot, ankle, and Achilles tendon, and a sport scientist both were aware of the concepts being examined in the questionnaire. A third translator without medical background ("naïve translator") was not familiar with the questionnaire's concepts. Written reports were made to indicate rationales for decision making, linguistic difficulties and problems with regards to content.

Step II: Considering the original VISA-A the resulting three German versions were synthesized during a consensus meeting of the three translators. A written report documented the procedure.

Step III: Back translation of the preliminarily VISA-A-G questionnaire was then conducted by two American English native speakers fluid in German but without medical background and blind to the original VISA-A.

Step IV: An expert committee was constituted including forward and back translators, a health professional, and a language professional. This panel developed the prefinal VISA-A-G version by consensus discussion with respect to semantic, idiomatic, experiential, and conceptual equivalence based on all previous material (original, target and back translated questionnaires including the respective reports).

Step V (pretesting): The prefinal questionnaire was filled out by a cohort of 35 male and 42 female persons (asymptomatic persons). These persons were then asked regarding their individual understanding of the questionnaire's items. The interviewer prepared a report on the test subjects, understanding of the items, and their decision making. Again the questionnaire was adapted respectively resulting in a final version.

Step VI: The final version of the VISA-A-G questionnaire and all reports and forms were then subjected to a review done by the developers of the VISA-A-G questionnaire for appraisal of the adaptation process.

The final VISA-A-G questionnaire was then subjected to psychometric testing including an analysis of reliability and validity in 30 Achilles tendinopathy patients and 79 asymptomatic people. Internal consistency was evaluated in 30 Achilles tendinopathy patients.

This was done following closely the procedure described in the original VISA-A report [8].

Test subjects: For reliability and validity testing four groups of test subjects were defined according to the status of their Achilles tendon. Group I included 15 preoperative Achilles tendinopathy patients selected prospectively from the own center. Group II consisted of 15 Achilles tendinopathy patients conservatively treated. These patients were recruited from review of the most recent charts in our sports medicine center. 48 Frankfurt/Main university pharmacology students (one class preparing for their examinations) without Achilles tendon complaints constituted group III. Group IV was formed by 31 members of three local running clubs serving as non injured but active controls. That population was recruited by direct contact during their practise.

Inclusion criteria for all four groups were: age over 18 years, willingness to take part in this project, and ability to give written informed consent. Contrasting to the original VISA-A publication [8] only patients suffering from unilateral Achilles tendinopathy were included (group I and II). Achilles tendinopathy was defined as tenderness of the midportion of the Achilles tendon 2 to 7 cm proximal to the Achilles tendon insertion and a history of load induced pain in this area [2-4]. This clinical diagnosis includes degenerative Achilles tendon lesions, Achilles tendon partial tears on a microscopic level, and paratendinosis or combinations of these. For the control groups (group III and VI) subjects with previous Achilles tendon injuries were included, if they did not complain any actual symptoms or functional deficiencies. Persons with inadequate compliance and pregnant or nursing women were excluded from participating in any of the four groups. Patients (group I and II) with bilateral symptoms, subtotal or complete Achilles tendon tears, insertional Achilles tendinopathy, Haglund's disease, and relevant injuries or previous surgery of the lower extremity, radicular and pseudoradicular induced pain were excluded.

To document the individual level of activity the ankle activity score which is a reliable, valid, and sensitive measure [18] was calculated for all test subjects.

For concurrent validity testing all participants (n = 109) completed the VISA-A-G questionnaire and Achilles tendinopathy severity was additionally rated by an orthopaedic surgeon specialized in Achilles tendon disorders (HL) using a generic tendon grading system [19] and a "classification system for the effect of pain on athletic performance" [20]. Even if the clinimetric properties of these tools are not formally validated so far they have been used as a standard for concurrent validity testing in previous VISA-A validation research [8,9,12]. Percy and Conochie categorized pain and function following surgery of Achilles tendon tears: "Excellent" means full function and no residual disability. "Good" is defined as full function with no real limitation of activities and minor pain. A "fair" result is represented by some limitation of activities. Severe weakness and marked limping indicates a "poor" result [19]. Curwin and Stanish assessed six degrees of reported exercise induced tendon pain and the level of sports performance: 1 = no pain and unrestricted level of sports performance; 2 = pain only with extreme exertion and unrestricted level of sports performance; 3 = pain with extreme exertion and 1 to 2 hours afterward and normal or slightly decreased level of sports performance; 4 = pain during and after any vigorous activities and somewhat decreased level of sports performance; 5 = pain during activity forcing termination and markedly decreased level of sports performance; 6 = pain during daily activities and unable to perform sport [20].

Besides this, VISA-A-G results for patients expected to present moderate symptoms (group II) and patients probably suffering the most severe symptoms (group I) were compared with the controls (group III and IV).

Next, the VISA-A-G ratings from this study were compared with the respective VISA-A group values presented in the original publication [8].

For reliability testing a subset of subjects were included. For test retest reliability all conservative patients (n = 15), all students (n = 48), and all joggers (n = 31) were evaluated by the VISA-A-G questionnaire two times within one week. Intertester reliability was assessed by comparing the results when both authors administered the VISA-A-G questionnaire independently in a one to seven days interval to five participants from the students and conservative Achilles tendinopathy group respectively.

### Statistical analysis

Statistics were performed using descriptive data analysis as mean, standard deviation and 95% confidence interval. The Kolmogorov-Smirnov test was applied to check out for normal distribution. Concurrent validity was calculated by Spearman's rank correlation coefficient between the VISA-A-G scores and the two standard scoring scales [19,20]. Comparison of the VISA-A-G group scores with the originial VISA-A [8] was performed using a two sample t-test since only means and standard deviations were available from the original report. Reliability testing was done by Spearman's rho, Inter-Class Correlation Coefficient (ICC) and Wilcoxon paired test for nonparametric data. Internal consistency for the total score was calculated using Cronbach's alpha. Significance level was set at p < 0.05.

## Results

### Cross-cultural adaptation of the VISA-A to the VISA-A-G questionnaire

With regards to content one of the forward translators had troubles to understand the meaning of the predetermined different answers and scorings to question 8 ("If you have no pain while undertaking Achilles tendon loading sports, for how long can you train/practise?"): If someone would have no sport related Achilles tendon pain, (s)he will be able to train as long as (s)he likes. Considering this, all persons free of Achilles tendon pain would consequently be expected to score 30 points. In this sense this question seemed to be irrelevant for patients suffering from Achilles tendinopathy. We discussed this problem with the developers of the original VISA-A questionnaire by E-mail who cleared this issue: if someone is just recovering from Achilles tendinopathy, (s)he may be on reduced training at first, say 15 minutes necessitating further answer categories (Karim Khan personal communication, 2009). Besides this there were no relevant problems reported from the three forward and also from the back translators and minor linguistic discrepancies could easily be resolved in the consensus and expert committee meeting respectively. Prefinal VISA-A-G questionnaire testing revealed some difficulties in comprehension of the questions 1 and 6. According to the Swedish translation [16] the boxes were marked with the related minutes/times to complete the final VISA-A-G version. Finally the developers approved the VISA-A-G questionnaire [see Additional file 1] and the cross-cultural adaptation process.

### VISA-A-G test population

The tested groups were not homogeneous comparing age (Table 1). Asymptomatic students (Group III) were significantly younger than the subjects in the three other groups (each p < 0.001) and preoperative patients were older than the joggers group (p = 0.032). The ankle activity mean score did not differ significantly (p = 0.733 to 0.999) between the four test groups (Table 1). Achilles tendinopathy severity as measured with the generic tendon grading system [19] and the "classification system for the effect of pain on athletic performance" [20] did not differ comparing the preoperative with the conservative patients, the students, and the joggers group respectively (each p = 0.000). There was no difference found between the jogger's and the student's groups (p = 0.864 and 0.998; Table 1).

**Table 1:** Age, outcome of the VISA-A-G, Ankle Activity Score [18] Achilles tendinopathy severity as measured with a generic tendon grading system [19] and the "classification system for the effect of pain on athletic performance"[20] for the study population.

	**Age [years]**			**VISA-A-G Score**			**Ankle Activity Score **[18]			**Percy & Conochie Score **[19]			**Curwin & Stanish Score **[20]		
	**Mean**	**SD**	**95% CI**	**Mean**	**SD**	**95% CI**	**Mean**	**SD**	**95% CI**	**Mean**	**SD**	**95% CI**	**Mean**	**SD**	**95% CI**
Preoperative Patients	47,8	11,4	41,5-54,1	44,9	14,2	37,0-52,7	4,6	1,5	3,8-5,4	1,5	0,5	1,2-1,8	5,3	0,7	4,9-5,7

Conservative Patients	44,6	14,0	36,9-52,4	73,1	13,5	65,6-80,5	5,2	1,6	4,3-6,1	2,3	0,8	1,8-2,7	3,3	1,2	2,7-4,0

Students	21,0	3,9	20,0-22,1	98,0	7,1	95,9-100,0	4,9	2,1	4,3-5,5	3,8	0,4	3,7-4,0	1,2	0,7	1,0-1,4

Joggers	39,3	11,7	35,0-43,6	99,2	2,0	98,5-99,9	4,7	0,5	4,5-4,9	3,7	0,5	3,6-3,9	1,3	0,4	1,1-1,4

SD = standard deviation; CI = 95% confidence interval.

### VISA-A-G score results

**Figure 1:**
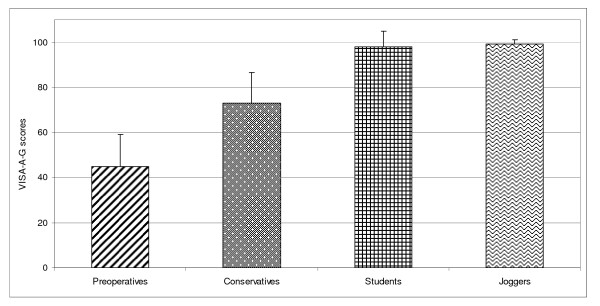
**Bar chart demonstrating the VISA-A-G mean scores (plus standard deviation) in relation to the tested groups**.

Related to controls (group III and IV) VISA-A-G scores for patients (group I and II) were significantly lower (p = 0.000). VISA-A-G questionnaire outcome was not different comparing the asymptomatic control groups III with IV (p = 0.347) while the symptomatic groups I with II differed significantly (p = 0.000). The preoperative and the conservative group each were significantly different from both control groups (each p = 0.000 Table 1, Figure 1).

### Validity testing

Comparing the VISA-A-G ratings from this study with the respective VISA-A group values presented in the original publication [8] revealed no significant differences comparing the healthy and the preoperative patient groups. Comparison between the "conservative" groups just reached the significance level (Table 2).

**Table 2:** Results of VISA-A-G ratings from the present study compared with the original English publication [8].

			**Present study**			
			**Healthy**		**Patients**	
			
			**Students** **98 ± 7**	**Joggers** **99 ± 2**	**Conservatives** **73 ± 142**	**Preoperatives** **45 ± 14**
		
		n	48	31	15	15
**Robinson** [**et al.** 8]	Healthy 96 ± 7	63	0.138			
	
	Joggers 98 ± 3	20		0.198		
	
	Conservatives 64 ± 17	45			0.050	
	
	Preoperatives 44 ± 28	14				0.905

**Figure 2:**
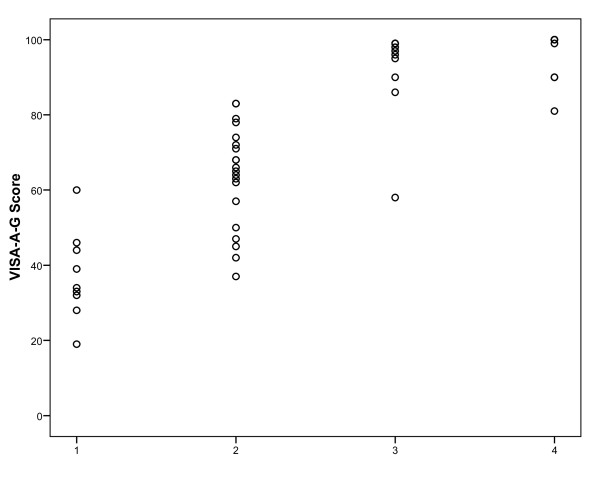
**Relation between VISA-A-G score and Achilles tendon grade of severity by Percy and Conochie **[19]. 1 = excellent (full function and no residual disability); 2 = good (no real limitation of activities); 3 = fair (some limitation of activities); 4 = poor (severe weakness and marked limp).

**Figure 3:**
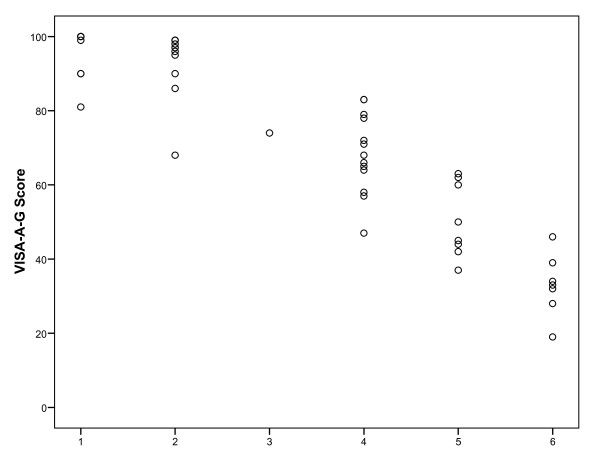
**Relation between VISA-A-G score and the "classification system for the effect of pain on athletic performance" by Curwin and Stanish **[20]. 1 = no pain and unrestricted level of sports performance; 2 = pain only with extreme exertion and unrestricted level of sports performance; 3 = pain with extreme exertion and 1 to 2 hours afterward and normal or slightly decreased level of sports performance; 4 = pain during and after any vigorous activities and somewhat decreased level of sports performance; 5 = pain during activity forcing termination and markedly decreased level of sports performance; 6 = pain during daily activities and unable to perform sport.

The VISA-A-G questionnaire correlated significantly with both tendon grading systems (concurrent validity). For the Percy and Conochie tendon classification [19] Spearman's rho was 0.95 (p = 0.000; Figure 2). Spearman's rho was determined to be         -0.95 (p = 0.000) when VISA-A-G questionnaire and the "classification system for the effect of pain on athletic performance" [20] were compared (Figure 3).

### Reliability testing

Reliability testing revealed no relevant difference when subsets of participants of the study were evaluated by the VISA-A-G questionnaire two times within one week (ICC = 0.60 to 0.97; Table 3). This was also true for reliability analysis of the individual test items 1, 2, 3, 4, 5, 7 and 8 (ICC = 0.65 to 0.89; Table 4). For question 6 ICC was 0.44 (p = 0.159). Interobserver agreement was excellent (n = 10; Pearson r = 0.97, p = 0.000; ICC = 0.99, p = 0.000).

**Table 3:** Results of reliability testing of the VISA-A-G questionnaire.

	**Spearman's rho**	**p**	**ICC**	**p**	**Wilcoxon**	**Cronbach's alpha**
Test-Retest Conservative Group (n = 15)	0.66	0.007	0.87	0.000	0.442	

Test-Retest Students Group (n = 48)	0.60	0.000	0.97	0.000	0.011	

Test-Retest Joggers Group (n = 31)	0.70	0.000	0.60	0.033	0.020	

Internal consistency Patients Group (n = 30)						0.74

ICC = Intraclass Correlation Coefficient.

**Table 4:** Results of reliability testing of the eight VISA-A-G items.

	**Spearman's rho**	**p**	**ICC**	**p**	**Wilcoxon**
Question 1	0.58	0.022	0.89	0.000	0.916

Question 2	0.89	0.000	0.89	0.000	0.942

Question 3	0.64	0.009	0.53	0.071	0.088

Question 4	0.65	0.009	0.69	0.022	0.565

Question 5	0.56	0.031	0.65	0.023	0.223

Question 6	0.23	0.411	0.44	0.159	0.681

Question 7	0.69	0.004	0.84	0.001	0.141

Question 8	0.65	0.009	0.75	0.007	0.279

ICC = Intraclass Correlation Coefficient.

## Discussion

Results of self-administered questionnaires in "populations divided by language or by culture" may systematically be biased if not equivalent in the original and target version [17]. Therefore it was necessary to adapt the VISA-A score also to a German version.

The most important finding of the present study is that the VISA-A score was successfully adapted to a German version and this is introduced. Reliability and validity have been tested showing moderate to excellent results.

Systematic stepwise translation and cross-cultural adaptation of the VISA-A questionnaire into a German version (VISA-A-G) was successfully performed. To be most accurate and comparable this process was performed by following the guidelines developed by Beaton et al. as close as possible [17]. Summarizing the results of the six stages of cross-cultural adaptation only question 8 caused some confusion to the forward and back translators and during the expert committee meeting as well. If someone has no sport related Achilles tendon pain and scores 0 min of sports ability, the reason not to train cannot be related to Achilles tendon pain but may be due to low general fitness. We discussed this point with the authors of the original VISA-A questionnaire by E-mail. Besides considering that different answering modalities have been introduced to enable pain free patients to score their actual level of Achilles tendon rehabilitation we were notified that "in practice" they "haven't had any problems with this" (Karim Khan personal communication, 2009).

During VISA-A-G questionnaire pretesting we realized that some subjects got into trouble marking the correct question 1 answering boxes. Analogue to the VISA-A-S cross-cultural adaptation [9] we facilitated filling out this question by inserting the minutes in each respective box in the final VISA-A-G version.

For the validation and reliability testing we implemented the methods which were described in the original publication [8]. Showing significant (p = -0.95 and 0.95) correlations between the VISA-A-G questionnaire and the two compared classification systems in all tested groups concurrent validity testing of the VISA-A-G questionnaire is considered to be excellent. Presenting ICC values from 0.60 to 0.97 also the reliability testing was proven to be "moderate" to "accurate" [21] with respect to each individual group of test subjects. It is unclear, why question 6 could not be proven to be sufficiently reliable. VISA-A-G questionnaire results are not biased by different observers. This is a result of the self administered and easy to use nature of the questionnaire. Face validity as assessed by the observers was good. From our experience with the testing procedure we feel that it can enable also postal explorations.

The authors of the original VISA-A questionnaire emphasize that it "is not a diagnostic tool" nor does it play a role in decision making for Achilles tendon surgery [8] and this statement is true also for the respective translations including the VISA-A-G questionnaire. Specific local pathologies (different foot and ankle disorders), central or peripheral sciatic nerve lesions have to been ruled out as differential diagnoses before administering one of the VISA-A questionnaires.

Included pathologies are different from previous work on this topic. Contrasting to the original VISA-A publication and to its Swedish translation [8,9] but according to the Italian translation [12] patients suffering from Haglund's disease and insertional tendinopathy were excluded. We believe that these are completely separate pathoanatomic entities [22] and its inclusion possibly may bias the results. Therefore the data presented for the validation and reliability testing of the VISA-A-G questionnaire are valid only for Achilles tendinopathy. A separate validation procedure is recommended for Haglund's disease and insertional Achilles tendinopathy patients. Also different from the original VISA-A publication [8] and from the Swedish translation [9] we excluded patients with bilateral Achilles tendon symptoms from participating in the study as this could possibly confound the answering behaviour.

The VISA-A questionnaire is a universally accepted instrument. In a Medline search 23 publications were found entering "VISA-A" in the "all fields" category. Of these eight were randomized and controlled trials and used the VISA-A questionnaire (n = 6) or the VISA-A-S (Swedish version) questionnaire (n = 2). There were two cross-cultural VISA-A adaptations [9,12]. Analyzing the methodological details, however, five of the randomized controlled investigations were performed (at least in part) in not English speaking countries and VISA-A questionnaires which are not cross-culturally adapted were used [10,11,13-15]. Even when published in high ranked and reviewed journals the results obtained from these studies are likely biased "as translation does not automatically provide a valid measure of another culture's health" [17]. The VISA-A score is developing to be the gold standard to determine the clinical severity of painful Achilles tendon lesions. Further cross-cultural adapted and validated versions of the VISA-A questionnaire are needed especially when international study groups from different language speaking countries cooperate and compare their results.

The present study explores data on concurrent validity testing of the VISA-A-G questionnaire and compares the results in groups of university students, uninjured joggers, conservatively treated, and preoperative patients with the published results from the original publication (VISA-A). Responsiveness including the minimal detectable change and the minimal clinically important difference have to be evaluated in future investigations.

## Conclusion

The VISA-A questionnaire was successfully cross-cultural adapted and validated for use in German speaking populations. The psychometric properties of the VISA-A-G questionnaire are similar to those of the original English and the Swedish version. It therefore can be recommended as a sufficiently robust tool for future measuring clinical severity of Achilles tendinopathy in German speaking patients.

## Competing interests

The authors declare that they have no competing interests.

## Authors' contributions

HL conceived the study, participated in its design, performed data acquisition, interpreted the data and drafted the manuscript. TN conceived of the study, participated in its design, performed data acquisition, analyzed and interpreted the data and helped to draft the manuscript. Both authors read and approved the final manuscript.

## Pre-publication history

The pre-publication history for this paper can be accessed here:



## Supplementary Material

Additional file 1 **Final version of the VISA-A-G questionnaire**. translated and cross-culturally adapted VISA-A-G questionnaire. Click here for file
