# Cross-cultural adaptation and validation of the VISA-A questionnaire for German-speaking Achilles tendinopathy patients

**DOI:** 10.1186/1471-2474-11-37

**Published:** 2010-02-26

**Authors:** Heinz Lohrer, Tanja Nauck

**Affiliations:** 1 Institute for Sports Medicine, Otto-Fleck-Schneise 10, D-60528 Frankfurt am Main; Germany

## Correction

After the work [1] was published in provisional form we realized a major error with regard to contents.

On page 4, in the first paragraph of the second column, the third sentence scores collected using different grading systems were compared. The difference "comparing the preoperative with the conservative patients, the students, and the joggers group respectively" was p = 0.000 for each individual comparison. Therefore in line eight and nine the term "...did not differ..." inverts this context. That phrase therefore has to be corrected to: **"...differed significantly..."**

The authors regret any inconvenience potentially caused by this error.

## Pre-publication history

The pre-publication history for this paper can be accessed here:

http://www.biomedcentral.com/1471-2474/11/37/prepub
