# Supplementary material for: Cross-cultural adaptation and validation of the VISA-A questionnaire for German-speaking Achilles tendinopathy patients
Source: BMC Musculoskelet Disord. 2009 Oct 30;10:134. doi: 10.1186/1471-2474-10-134 (PMC2776582; doi:10.1186/1471-2474-10-134)
Supplement: Additional file 1 — Final version of the VISA-A-G questionnaire. translated and cross-culturally adapted VISA-A-G questionnaire. [file 1471-2474-10-134-S1.doc]

**Name Vorname Geburtsdatum Datum**

**VISA-A-G Fragebogen**

**Bei diesem Fragebogen bezieht sich der Begriff „Schmerz“ speziell auf Schmerzen der Achillessehnenregion**

**1.** Für wie viele Minuten verspüren Sie nach dem ersten Aufstehen ein Steifigkeitsgefühl in der Achillessehnenregion?

| **100 min** | **90 min** | **80 min** | **70 min** | **60 min** | **50 min** | **40 min** | **30 min** | **20 min** | **10 min** | **0 min** |
| --- | --- | --- | --- | --- | --- | --- | --- | --- | --- | --- |

100 Min. 0 Min.

0 1 2 3 4 5 6 7 8 9 10 ...…...PUNKTE

**2.** Nachdem Sie für den Tag aufgewärmt sind, haben Sie Schmerzen, wenn Sie die Achillessehne über der Kante einer Treppenstufe dehnen? (Knie gestreckt halten)

Starker, Kein

heftiger Schmerz □ □ □ □ □ □ □ □ □ □ □ Schmerz

0 1 2 3 4 5 6 7 8 9 10 ……...PUNKTE

**3.** Nachdem Sie 30 Minuten auf ebenem Untergrund gegangen sind, haben Sie in den darauf folgenden 2 Stunden Schmerzen? (Wenn Sie wegen Schmerzen nicht auf ebenem Untergrund 30 Minuten gehen können, kreuzen Sie bei dieser Frage 0 an.)

Starker, Kein

heftiger Schmerz □ □ □ □ □ □ □ □ □ □ □ Schmerz

0 1 2 3 4 5 6 7 8 9 10 ……...PUNKTE

**4.** Haben Sie Schmerzen, wenn Sie mit normaler Geschwindigkeit die Treppe heruntergehen ?

Starker, Kein

heftiger Schmerz □ □ □ □ □ □ □ □ □ □ □ Schmerz

0 1 2 3 4 5 6 7 8 9 10 ……...PUNKTE

**5.** Haben Sie Schmerzen während oder unmittelbar nachdem Sie 10 (einbeinige) Zehenstände auf einer flachen Unterlage ausgeführt haben?

Starker, Kein

heftiger Schmerz □ □ □ □ □ □ □ □ □ □ □ Schmerz

0 1 2 3 4 5 6 7 8 9 10 ……...PUNKTE

**6**. Wie oft können Sie ohne Schmerzen auf einem Bein hüpfen?

| **0 mal** | **1 mal** | **2 mal** | **3 mal** | **4 mal** | **5 mal** | **6 mal** | **7 mal** | **8 mal** | **9 mal** | **10 mal** |
| --- | --- | --- | --- | --- | --- | --- | --- | --- | --- | --- |

0 1 2 3 4 5 6 7 8 9 10 ……...PUNKTE

**7.** Üben Sie derzeit Sport oder andere körperliche Aktivitäten aus?

□ 0 _ Nein, gar nicht; wegen der Achillessehnensymptome

□ 4 _ Verändertes Trainings ± Wettkampfverhalten verglichen mit dem Zustand vor Beginn der Symptome

□ 7 _ Volle Trainings ± Wettkampfbelastung, aber nicht auf dem gleichen Niveau als vor Beginn der Symptome

□ 10 _ Voll belastbar

**oder**

Trainings ± Wettkampfbelastung auf dem gleichen oder auf höherem Niveau als vor Beginn der Symptome

…… PUNKTE

**8.** Bitte beantworten Sie entweder A, B oder C dieser Frage

- Wenn Sie bei der Durchführung achillessehnenbelastender Sportarten keine Schmerzen empfinden, füllen Sie bitte nur Frage 8a aus.
- Wenn Sie bei der Durchführung achillessehnenbelastender Sportarten Schmerzen empfinden, die Sie aber nicht zum Belastungsabbruch der Aktivität zwingen, füllen Sie bitte nur Frage 8b aus.
- Wenn Sie bei der Durchführung achillessehnenbelastender Sportarten Schmerzen empfinden, die Sie zum Abbruch der Aktivität zwingen, füllen Sie bitte nur Frage 8c aus.

**8a.** Wie lange können Sie trainieren/üben, wenn Sie bei der Durchführung achillessehnenbelastender Sportarten keine Schmerzen empfinden?

0 1-10 Min. 11-20 Min. 21-30 Min. >30 Min.

□ □ □ □ □

0 7 14 21 30 …… PUNKTE

**ODER**

**8b**. Wie lange können sie trainieren/üben, wenn Sie bei der Durchführung achillessehnenbelastender Sportarten Schmerzen empfinden, die Sie aber nicht zum Belastungsabbruch der Aktivität zwingen?

0 1-10 Min. 11-20 Min. 21-30 Min. >30 Min.

□ □ □ □ □

0 4 10 14 20 …… PUNKTE

**ODER**

**8c.** Wie lange können sie trainieren/üben, wenn Sie bei der Durchführung achilles-sehnenbelastender Sportarten Schmerzen empfinden, die Sie zum Abbruch der Aktivität zwingen?

0 1-10 Min. 11-20 Min. 21-30 Min. >30 Min.

□ □ □ □ □

0 2 5 7 10 …… PUNKTE

**Gesamt-Score (Punkte/100) ……... %**
